# OsLMP1, Encoding a Deubiquitinase, Regulates the Immune Response in Rice

**DOI:** 10.3389/fpls.2021.814465

**Published:** 2022-01-18

**Authors:** Jing Sun, Wenzhong Song, Yuan Chang, Yanwei Wang, Tiegang Lu, Zhiguo Zhang

**Affiliations:** Biotechnology Research Institute, Chinese Academy of Agricultural Sciences, Beijing, China

**Keywords:** rice, lesion mimic, epigenetic modification, histone deubiquitination, immune response, SA

## Abstract

Lesion mimic mutants have become an effective material for understanding plant-microbe interactions and the immune mechanism in plants. Although many mechanisms responsible for the lesion mimic phenotype have been clarified in plants, the mechanism by which lesion mimic is regulated by posttranslational modification remained largely elusive, especially in rice. In this study, a mutant with the lesion mimic phenotype was obtained and named *lmp1-1*. Physiological measurements and quantitative real-time PCR analysis showed that the defense response was activated in the mutants. Transcriptome analysis showed that the phenylalanine ammonia lyase (PAL) pathway was activated in the mutant, causing the accumulation of salicylic acid (SA). The results of mapping based cloning showed that *OsLMP1* encodes a deubiquitinase. OsLMP1 can cleave ubiquitination precursors. Furthermore, OsLMP1 epigenetically modifies SA synthetic pathway genes by deubiquitinating H_2_B and regulates the immune response in rice. In summary, this study deepens our understanding of the function of OsLMP1 in the plant immune response and provides further insight into the relationship between plants and pathogenic microorganisms.

## Introduction

Rice, one of the main food crops in the world, supports half of the world’s population. Pathogenic microorganism invasion often decreases grain weight and the seed setting rate, which seriously affects the yield and quality of crops. Improving the disease resistance of crops itself is one of the most environmentally friendly, economic and effective ways to defend against diseases and is of great significance for maintaining stable and high crop yields (Vejan et al., 2016). Lesion mimic is a phenomenon in which local cell necrosis occurs spontaneously in plants without invasion by external pathogens. Most lesion mimic mutants show increased disease resistance to at least one microorganism; thus, they are good materials with which to study the mechanism of the plant defense response (Wu et al., 2008).

As an increasing number of disease resistance genes have been cloned and their functions elucidated, the mechanism responsible for the lesion mimic phenotype can be summarized by the following four observations. First, the resistance genes nucleotide binding site/leucine rich repeats (*NBS-LRRs*) were found to lead to the disorder of signaling pathways in the defense response, triggering the irreversible death of cells and eventually the occurrence of lesion mimic in plants. Mutations in *rice* necrotic leaf sheath1 (*NLS1*) (Tang et al., 2011), *maize* resistance protein (*RP1*) (Sun et al., 2001), and *Arabidopsis* Toll/interleukin-1 receptor (TIR)-NBS (*TN13*) (Cai et al., 2021) activate the expression of defense genes and thus cause high levels of salicylic acid (SA) accumulation and leaf necrosis spots. Second, blockade of the synthesis of some intermediate metabolic products or the accumulation of these products directly or indirectly leads to the lesion mimic phenotype. Due to mutations in rice lesion initiation 1 (*rlIN1*) (Sun et al., 2011) and *Arabidopsis* mosaic death1 (*MOD1*) (Mou et al., 2000), cell death occurs. Third, activation of the programmed cell death (PCD) pathway in plants results in the lesion mimic phenotype. *Arabidopsis* lesions *s*imulating *d*isease resistance 1 (*LSD1*) (Kaminaka et al., 2006), which encodes a zinc finger protein, promotes the production of excessive hydrogen peroxide in mutants and negatively regulates the PCD pathway. Fourth, the occurrence of lesion mimic is accompanied by changes in plant hormones, such as SA (Mosher et al., 2010; Singh et al., 2018), jasmonic acid (Feng et al., 2020), ethylene (Bouchez et al., 2007) and abscisic acid (ABA) (Mosher et al., 2010); Among these hormones, increasing the SA content significantly improves disease resistance (Singh et al., 2018). Most of the abovementioned regulatory proteins are disease-resistant NBS-LRRs, metabolic enzymes and transcription factors and drive lesion mimic at the transcriptional level and rarely at the posttranscriptional and translational levels.

Ubiquitination and deubiquitination are a kind of posttranslational modification. Deubiquitinases are some of the most abundant proteases in the ubiquitin system (Hu, 2012). Based on their amino acid sequences, deubiquitinases can be divided into cysteine proteases and metalloproteinases. Cysteine proteases include four subfamilies: ubiquitin-specific proteases (UBPs), ubiquitin C-terminal hydrolase (UCH), ovarian tumor-related proteases (OTUs), Machado Joseph domain-containing proteases (MJDs), and the Jab1/MPN/Mov34 (JAMM) family (Amerik and Hochstrasser, 2004). Deubiquitination is involved in the regulation of not only important life activities in animals and yeast (Clague et al., 2013) but also plant growth and development (Katsiarimpa et al., 2014). Upon the mutation of *osubp6 in Oryza sativa var. japonica cv. Dongjin*, the seedlings grew slowly but then showed normal growth at a later stage (Moon et al., 2009). Seed development and heading date were affected in an *atubp26* mutant (Luo et al., 2008). Further study showed that the *AtUBP26* mutation caused the accumulation of ubiquitin H_2_B, which indirectly influenced the levels of H_3_K_27_me3 and H_3_K_36_me3 at the flowering locus C (FLC) site, and the transcription of FLC was inhibited in the *atubp26* mutant (Schmitz et al., 2009). H_2_B-Ub stimulates Dot1L activity and the contacts mediated by Dot1L and the H4 tail induce a conformational change in the globular core of histone H3 that reorients K79 from an inaccessible position, thus enabling this side chain to insert into the active site in a position primed for catalysis (Worden et al., 2019). *AtUBP27* is located in mitochondria, and its mutation does not influence mitochondrial morphology. However, mitochondrial morphology did change when *AtUBP27* was overexpressed (Pan et al., 2014). In addition, UBP/UCH family members participate in multiple signaling pathways. UBP6 participates in the Ca^2+^ signaling pathway (Moon et al., 2005). UBP24 deubiquitination modulated the responses to ABA and salt stress (Zhao et al., 2016). Overexpression of *UCH1* in *Arabidopsis* restored the auxin insensitivity phenotype caused by the mutation of *axr1-3* and *axr2-1*, indicating that UCH1 may be involved in the auxin signaling pathway (Yang et al., 2007). In conclusion, deubiquitination not only regulates the growth and development of plants but also plays a very important role in mediating photomorphogenesis, hormone signal transduction, and abiotic stress responses. These diverse functions highlight the importance of deubiquitination in biological processes; however, the immune response induced by deubiquitination remained largely elusive, especially in rice.

In the past decade, the studies had been established a role for epigenetic mechanisms in plant–pathogen interactions. Kong et al. (2017) found that the cytoplasmic effector PsAvh23 produced by the soybean pathogen *Phytophthora sojae* plays as a regulatory factor of histone acetyltransferase (HAT) in plants. PsAvh23 interfered with the association of ADA2, subunit of the HAT complex SAGA and disrupts the catalytic activities of ADA2 module. Thus, PsAvh23 regulated ADA2 module by suppressing H3K9 acetylation and improved plant susceptibility. PsAvh52 as an early-induced RxLR effector from the soybean root rot pathogen, *P. sojae*, interacted with GmTAP1 (Li et al., 2018). During early infection, PsAvh52 caused GmTAP1 to relocate into the nucleus and made histones H2A and H3 acetylation, thereby promoting susceptibility to *P. sojae*. In the absence of pathogen, GmTAP1 remained confined to the cytoplasm and did not modify plant susceptibility. Recent, PICI1, encoding a deubiquitinase, plays as an immunity hub for PTI and ETI in rice (Zhai et al., 2021). PICI1 is targeted for degradation by blast fungal effectors, including AvrPi9, to dampen PTI. Nucleotide-binding domain, leucine-rich-repeat-containing receptors (NLRs) in the plant immune system, such as PigmR, protect PICI1 from effector-mediated degradation to reboot the methionine-ethylene cascade. Although generational defense priming has only been in a few cases using model plants, the fine regulation between pathogenic microorganisms and plants needs to be further explored.

To study the immune response mechanism in rice, a lesion mimic mutant was obtained and named *lmp1-1*. In this study, *OsLMP1* was cloned by a map-based cloning strategy. A series of assays verified that *OsLMP1*, which encodes a deubiquitinase, epigenetic modifies H_2_B and regulates the plant immune response. This study broadens the function of deubiquitination in rice and deepens our understanding of the role of epigenetic modification in plant disease resistance.

## Materials and Methods

### Plant Materials and Growth Conditions

The *lmp1-1*, wild-type (*Nipponbare*), and *OsLMP1* complementation lines and *OsLMP1* knockout lines were planted in an experimental field under normal growth conditions at Langfang station in summer (22°C, 16 h light/8 h dark) and in Sanya in winter (22°C, 16 h light/8 h dark). The *lmp1-1* and wild-type lines were crossed to determine the inheritance law in the F_2_ progeny.

### Map-Based Cloning of *OsLMP1*

A genetic mapping population was constructed using *lmp1-1* as the recipient and the *Indica* variety *Dular* as the donor with wide compatibility. Among the F_2_ progeny, segregated plants with the lesion mimic phenotype were selected for coarse and fine mapping. *SNP* and indel molecular markers were designed based on whole-genome differences between *Dular* and *Nipponbare*. The candidate genes within the positioning interval were predicted, amplified and sequenced.

### Complementation Experiment

A complementation experiment was constructed as follows. First, the full coding sequence of *OsLMP1* was amplified and ligated into the *p*Cambia2300 vector. The coding sequence was driven by maize ubiquitin1. The fused complementation vector (*OsLMP1-p*Cambia2300) was transferred to the *Agrobacterium tumefaciens* AGL1 strain. *NPTII* was selected as the selectable marker. Calli of the *lmp1-1* strain were infected by the strain containing *OsLMP1-p*Cambia2300. The positive plants were identified by PCR amplification and sequencing (Hiei et al., 1994).

### Creation of the *OsLMP1* Knockout Lines

The CRISPR/Cas9 method was used to knock out *OsLMP1* with sgRNA designed to target *OsLMP1*. A single sgRNA was designed and inserted in the BGK03 vector containing Cas9, which was introduced into *Agrobacterium strain* AGL01 and transformed into *Nipponbare calli*. In total, twenty independent lines transfected with the sgRNA were obtained. Sequence alignment showed that *OsLMP1* deletion lines had been obtained.

### qRT-PCR Analysis

Total RNA was extracted from the *lmp1-1*, wild-type and complementation lines at the later tiller stage using TRIzol RNA reagent. Total RNA (1.5 μg) from each sample was reverse-transcribed with an oligo(dT) primer and Ace enzyme (Toyobo, Japan) according to standard procedure. The cDNA solutions were diluted at least 10-fold. The amplification procedure was as follows: an initial 94°C denaturation step for 5 min, followed by 25–30 cycles of 94°C for 30 s, 56°C for 20 s, and 72°C for 20 s and a final 72°C extension step for 10 min. OsActin1 was selected as a control. Pathogenic genes were selected for further analysis. The expression pattern of *OsLMP1* was determined in root, leaf, panicle, leaf sheath, and stem tissues at the seedling and flowering stages. Relative expression was calculated using the ΔΔ^CT^ method (Livak and Schmittgen, 2001). Wild-type leaves at the later tiller stage were treated with rice bacterial blight (PXO99). Tissues 2 cm from the incision were sampled every 4 h. The untreated wild type (mock) was selected as control.

### DAB Staining

The *lmp1-1* and wild-type leaves at the flowering stage were stained with DAB dye (Jubany-Mari et al., 2009), decolorized using ethanol, and then photographed.

### Measurement of the H_2_O_2_ Content

The H_2_O_2_ content was measured based on the protocol used by Zhang et al. (2013). Briefly, 2 g of fresh leaves from the *lmp1-1* and wild-type lines at the flowering stage were collected, mixed with acetone at a ratio of 1:1 and ground with a small amount of quartz sand to form a homogenate. The sample was centrifuged at 12,000 × *g* for 5 min. One milliliter of the supernatant was obtained and mixed with 5 ml of Ti(SO4)_2_ and 0.2 ml of 19% ammonia to make a precipitate. The precipitate was washed with acetone at least 3–5 times until the green pigment was removed. The resulting pellet was dissolved in 5 ml of 2 M H_2_SO_4_. The H_2_O_2_ content was calculated according to a standard curve made with H_2_O_2_ ranging from 0 to 10 mM.

### Subcellular Localization

The full *OsLMP1* coding sequence without its termination codon was amplified using PCR and ligated with PAN580 to generate 35S:*OsLMP1*:*GFP*. The fusion construct (35S:*OsLMP1*:*GFP*) and pMcherry (35S:OsMADS3:RFP, as a control) were transiently transformed into rice protoplasts extracted from 14-day-old seedlings by the PEG transformation method (Zhang et al., 2011). The pMcherry construct (35S:OsMADS3:RFP) has been described by Gao et al. (2013). After 16 h at 28°C in the dark, the transformed protoplasts were observed using confocal laser scanning microscopy (Leica TCS SP2) (Chen et al., 2006). Then observed with a 20× mirror and GFP fluorescence images were taken with a 488-nm argon laser and a 505–530-nm bandpass emission filter. Analyzed with ZEN 2009 Light Edition software, Scale bar = 5 μm.

### GUS Staining

The 2K promoter of *OsLMP1* was amplified and ligated with the *p*Cambia1391Z vector. The fusion vector (OsLMP1-1391Z) was transferred to the *A. tumefaciens* AGL1 strain. The strain was transformed to *Nipponbare* calli. The positive plants were stained, and tissue expression patterns were determined using the GUS staining method (Jefferson et al., 1987).

### Transcriptome Analysis

Total RNA was extracted from both the wild-type and *lmp1-1* lines at the later tiller stage (about 2 months growth period) using TRIzol reagent, and the RNA was sent to Novogene for transcriptome analysis. Each sample was assayed in triplicate. The transcriptome data were deposited at NCBI^1^.

### Deubiquitinase Activity Assay

A deubiquitinase activity assay was performed as described by Shi et al. (2019). The ubiquitinated OsUBQ1 (LOC_Os03g13170) and polyubiquitinated OsUBQ10 (LOC_Os02g06640) proteins from rice were selected as substrates (Moon et al., 2009). Recombinant GST-OsLMP1, GST-OsLMP1-m (in which an “A” base was inserted as position 2,604, generating a truncated protein), OsUBP10, and OsUBP1 plasmids were constructed and co-transformed into *Escherichia coli* Transetta (DE3). Expression of the proteins was induced by IPTG at 23°C for 8 h. Total proteins were extracted using a Millipore ProteoPrep kit and then boiled at 95°C for 10 min. After electrophoresis, the proteins were transferred to a PVDF membrane and then incubated with primary antibodies. The antibodies used in this study were anti-GST (CWBio) and anti-ubiquitin (Abcam). Signals were detected using a Pierce ECL Plus Western Blotting Detection Kit and visualized with an imaging system.

### Western Blot Analysis

Total histones were extracted from the leaf tissues of the wild-type, lmp1-1, crisp-m1, and complementation lines using a Millipore ProteoPrep kit at the later tiller stage. Total histones were boiled at 95°C for 10 min before immunoblotting. After electrophoresis, the proteins were transferred to a PVDF membrane and then incubated with the primary antibodies. Anti-H2B (1:1000) was purchased from Abcam (ab1790), and anti-H2B-ubi (1:1000) (Lys 120) was purchased from Active Motif (39623). Anti-H3 (ab1791) (1:2000), anti-H3K4me2 (07-030) (1:2000), anti-H3K36me2 (07-369) (1:2000), anti-H3K4me3 (07-73) (1:2,000), anti-H3K9me2 (05-1354) (1:2000), anti-H2A (ABE327) (1:1000), and anti-monoubiquitinated H2A (clone E6C5) (1:1000) were purchased from Abcam or Millipore. Signals were detected using a Pierce ECL Plus Western Blotting Detection Kit and visualized with an imaging system.

### ChIP-qPCR Analysis

ChIP was conducted as follows (Clontech, cat# 640166). First, cell nuclei were isolated from wild type seedlings, respectively, at the 5th leaf stage cross-linked with formaldehyde, and sonicated to shear the chromatin to fragment with an average size of 0.2–1.0 kb. The samples were incubated with protein A agarose beads (40 μl; 16–157, Millipore). Then, the resulting mixture was incubated at 4°C overnight with anti-H_2_B-ubi (Lys 120) combined with protein A agarose beads. The product was washed successively with high-salt NaCl, LiCl, and TE buffers. The washed samples were digested by proteinase K. The DNA fragments were cleaned up using a PCR DNA purification kit (Qiagen). The resulting DNA was analyzed by qRT-PCR using designed primers (Supplementary Table 6). The control was selected using protein A agarose beads incubated with chromatin samples in the absence of anti-H_2_B-ubi (Lys 120) antibody. The experiment was repeated three times. OsActin1 was used as negative control.

### Blast and Bacterial Blight Resistance Experiments

Bacterial blight strain PXO99, C1, C2, and C6 were used to infect the *lmp1-1* plants and wild type, respectively (Wang et al., 2014). *Magnaporthe oryzae* (*M. oryzae*) strain Dao72 was used to infect the *lmp1-1* plants and wild type (Wang et al., 2017a). The infected results were evaluated 1 weeks after inoculation.

### Measurement of the Salicylic Acid Content

Flag leaves (0.3 g) at the flowering stage (about 3 month period) from the wild-type and *lmp1-1* were prepared. SA was extracted and quantified as described (Chen and Jastreboff, 1995).

### Statistical Analyses

The experiments were repeated at least three times. The mean standard deviations are shown in the figures. Significant differences based on Student’s *t*-test are marked with asterisks (*P < 0.05, ^**^P < 0.01).

### Primer and Gene Sequences

The primers were designed according to Primer3 Input (version 0.4.0)^2^. The primers used are shown in Supplementary Table 6.

## Results

### Identification of the *lmp1-1* Mutant

**FIGURE 1:**
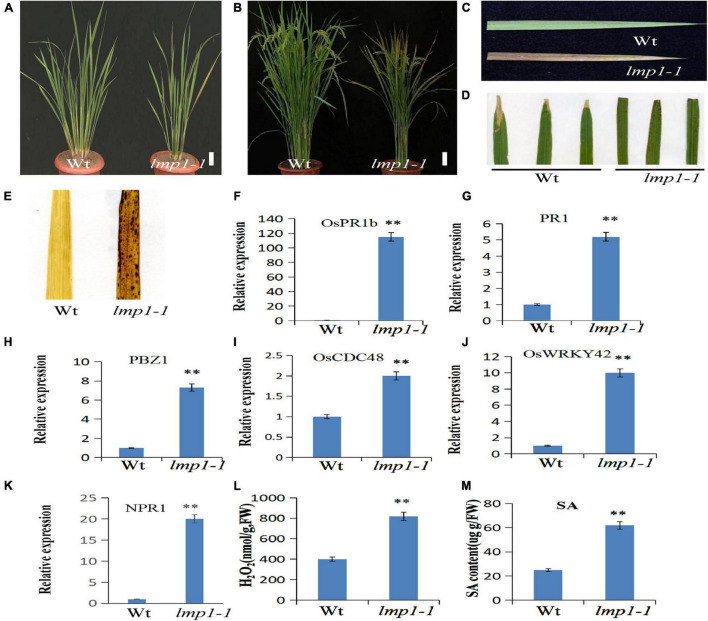
Morphological characterization of the *lmp1-1* and wild-type lines. **(A)** Phenotype comparison of field-grown wild-type (wt) and *lmp1-1* lines at the later tiller stage (bar = 5 cm). **(B)** Phenotype comparison of field-grown wt and *lmp1-1* lines at the flowering stage (bar = 5 cm). **(C)** Leaves collected from wt and *lmp1-1* plants shown in panel **(A)**, which also shows the lesion mimic phenotype in *lmp1-1*. **(D)** Enhanced resistance of *lmp1-1* to rice bacterial blight (PXO99). **(E)** Detection of accumulated H_2_O_2_ in wt and *lmp1-1* leaves at the flowering stage using DAB staining. **(F–K)** The expression levels of pathogenic genes in wt and *lmp1-1* plants at the later tiller stage were detected using qRT-PCR (*n* = 3). **(L)** The H_2_O_2_ content of wt and *lmp1-1* plants at the flowering stage was measured (*n* = 3). **(M)** The SA content of wt and *lmp1-1* plants at the flowering stage was measured (*n* = 3). The significant differences were marked with asterisks based on student’s *t*-test (^**^P < 0.01).

Previously, we constructed a rice T-DNA insertion population and obtained ∼100 lesion mimic mutants at different developmental stages (Wan et al., 2009). To study the mechanism of the immune response in rice, a lesion mimic phenotype mutant, named *lmp1-1*, was selected for further study. The *lmp1-1* plants began to exhibit necrotic spots on their leaves at the early tiller stage and showed a lesion mimic phenotype at the late tiller stage (Figure 1A). At the flowering stage, the leaves of *lmp1-1* plants became completely brown (Figures 1B,C).

A cross test between *lmp1-1* and wild-type plants showed that the segregation ratio in the F_2_ progeny of wild-type (156) and mutant-like (50) plants reached 3:1 (*x*^2^ = 0.81, *x*^2^_0.05_,_1_), indicating that *lmp1-1* is controlled by a single recessive nuclear locus.

The qRT-PCR analysis at the later tiller stage showed that the expression levels of pathogenesis-related genes, such as OsWRKY42, OsPR1b, and PBZ1, were significantly increased in the mutants (Figures 1F–K). An infection experiment at the flowering stage using four bacterial blight strains and one *M. oryzae* strain showed that the *lmp1-1* t plants were more resistant to the bacterial blight strain and *M. oryzae* strain than the wild-type plants, similar to most of the lesion mimic mutants (Figure 1D and Supplementary Figure 1).

### Disease Resistance in the *lmp1-1* Plants Is Associated With H_2_O_2_ Accumulation

Lesion mimic is often accompanied by the accumulation of reactive oxygen species, H_2_O_2_ (Tang et al., 2011). H_2_O_2_ in leaves can be stained with the dye 3,3′–diaminobenzidine (DAB) (Jubany-Mari et al., 2009). A DAB staining assay in mature leaves at the flowering stage showed that the leaves of *lmp1-1* plants were more deeply stained than those of the wild-type plants, indicating that more H_2_O_2_ had accumulated in the mutants (Figure 1E). Measurement of the H_2_O_2_ content in the leaves also verified this result (Figure 1L). Thus, we speculated that H_2_O_2_ accumulation in the mutants activated the defense response and caused the lesion mimic phenotype.

### Map-Based Cloning of OsLMP1

**FIGURE 2:**
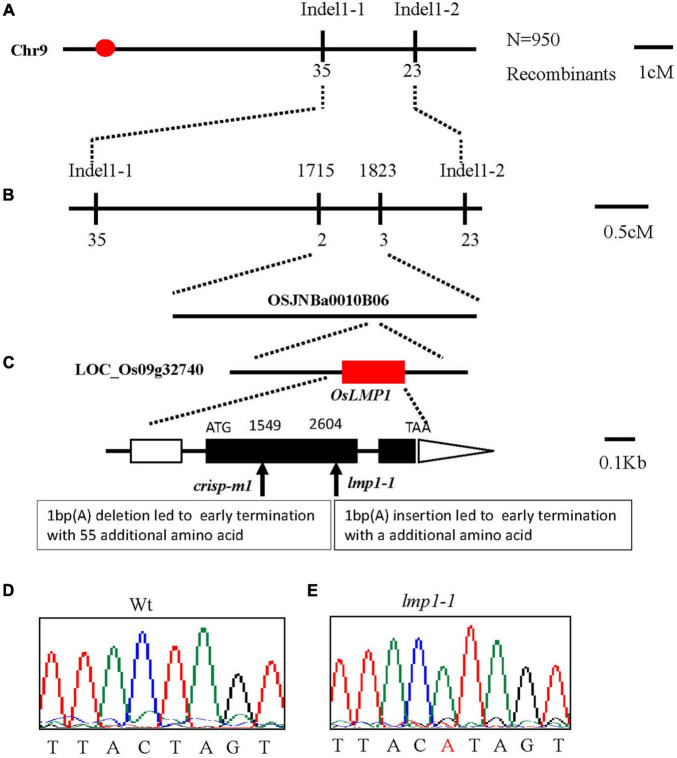
Map-based cloning of *OsLMP1*. **(A)** The OsLMP1 locus was mapped to a 3-cM interval on chromosome 9L between indel markers *Indel1-1* and *Indel1-2*. **(B)** The location of the OsLMP1 locus was narrowed down to a 110-kb region between indel markers 1,715 and 1,823 in the BAC clone OSJNBa0010B06 using 950 F_2_ homozygous mutant plants. **(C)** Eight open reading frames (ORFs) were predicted in the mapped region. Structure of OsLMP1. ATG, TAA: start and stop codons, respectively. Boxes: exons. Lines between black boxes: introns. One single nucleotide insertion results in a frame shift mutation in *lmp1-1*. The deletion site in crisp-m1 is labeled. The black box denotes the coding sequence from ATG to TAA. **(D,E)** Comparison of the mutant site of LOC_Os09g32740 between the wild-type **(D)** and *lmp1-1* mutant **(E)**. The inserted base is labeled in red in **(E)**.

The *lmp1-1* mutant did not cosegregate with the hygromycin gene, which indicated that the mutation may have originated from somatic variation induced by tissue culture. To clone *OsLMP1*, we first constructed an F_2_ mapping population (*lmp1-1*x*Dular*). Using map-based cloning, *OsLMP1* was roughly mapped to the region between *Indel1-1* and *Indel1-2* on chromosome 9 using 50 F_2_ mutant-like plants (Figure 2A). Fine positioning using 950 plants showed that *OsLMP1* was localized within 110 kilobase pairs (kbp) (Figure 2B). This region contains eight open reading frames. All the genes were sequenced, and a mutation in LOC_Os09g32740 was identified in the *lmp1-1* mutant (Figure 2C). An insertion of one base (“A”) at position 2,604 of the second exon in *lmp1-1* eventually led to early translation termination and an additional amino acids (Figures 2D,E).

**FIGURE 3:**
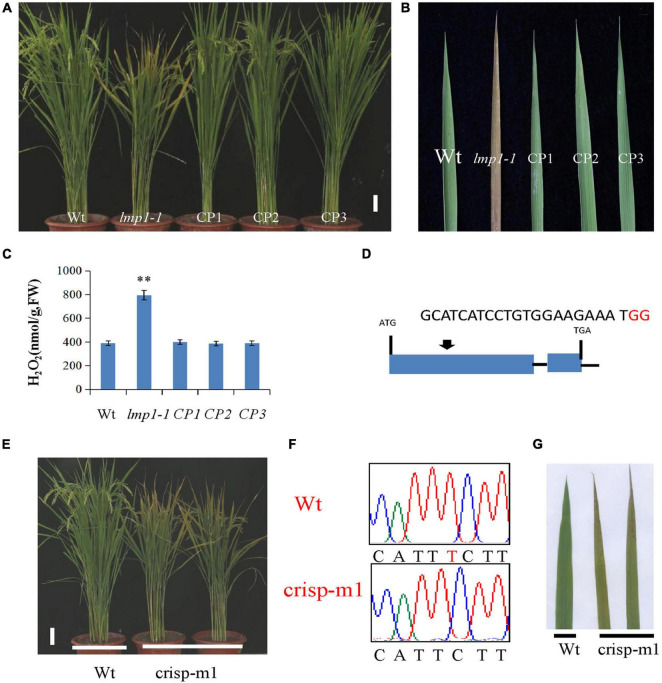
Verification of *OsLMP*1 by complementation assay and gene editing technology. **(A)** A complementation assay was conducted using full-length OsLMP1 cDNA. Phenotype comparison of field-grown wt, *lmp1-1*, and complementation lines (CP1–3) at the flowering stage (bar = 5 cm). **(B)** Leaves from the wt, *lmp1-1*, and complementation (CP1–3) lines collected from the plants shown in panel **(A)** show that complementation restored the lesion mimic phenotype at the flowering stage. **(C)** The H_2_O_2_ content of the wt, *lmp1-1*, and complementation (CP1–3) lines was measured at the flowering stage (*n* = 3). The significant differences were marked with asterisks based on student’s *t*-test (**P < 0.01). **(D)** Schematic map of the genomic region of *OsLMP1* and the sgRNA target site; the arrow shows the sgRNA target site on the *OsLMP1* genomic sequence, and the PAM motif (NGG) is shown in red. Blue boxes indicate OsLMP1 exons, and black lines indicate introns. **(E)** Phenotype comparison of the wt and crisp-m1 homozygous mutants at the flowering stage (bar = 5 cm). **(F)** The deleted base (1,549) is labeled in red in the wt. **(G)** Comparison of the wt and crisp-m1 leaves shows that crisp-m1 has the lesion mimic phenotype at the flowering stage.

A complementation vector was constructed by fusing the LOC_Os09g32740 coding sequence with *p*Cambia2300. The *A. tumefaciens strain* containing *p*Cambia2300-*OsLMP1* was transformed into the *lmp1-1* calli. The regenerated lines were observed with the naked eye and detected by PCR amplification (Figure 3A). Ten positive lines showed restoration of the normal leaf color (Figure 3B). The expression level of OsLMP1 in the complementation lines was restored to the expression level in the wild-type plants (Supplementary Figure 2). Measurement of the H_2_O_2_ contents in the leaves of the complementation lines (CP1–CP3) showed restoration to the wild-type levels (Figure 3C).

In addition, an allelic mutant named *crisp-m1* was created by CRISPR-Cas9 technology with the deletion of a single base (“T”) at position 1549 in the second exon, which led to early termination and an additional 55 amino acids (Figures 2C, 3D,F). The *crisp-m1* plants also showed the lesion mimic phenotype at the flowering stage (Figures 3E,G), which was consistent with the observations of *lmp1-1* plants. Overall, the complementation experiment and the allelic line verified that *OsLMP1* is responsible for the lesion mimic phenotype.

### Bioinformatics Prediction and Expression Pattern of *OsLMP1*

BLASTP sequence analysis with the *NCBI* database showed that *OsLMP1* encodes a ubiquitin-specific protease containing a zinc finger motif at its N-terminus from amino acids (aa) 132 to 190 and a C-terminal hydrolase catalytic C19 domain from aa 226 to 1,051. Peptidase C19 contains ubiquitin hydrolases, which are intracellular peptidases that remove ubiquitin molecules from polyubiquitinated peptides by the cleavage of isopeptide bonds. The ubiquitin/proteasome system is responsible for most protein turnover in mammalian cells, and with over 50 members, the C19 family is one of the largest families of peptidases in the human genome (Piao et al., 2015). We performed cluster analysis of UCHs in *Arabidopsis* and *rice.* OsLMP1 shows low homology with other UCH family genes in rice and is most closely related to *AtUBP1* and *AtUBP2*, with a sequence homology of 23.59% and 26.23% in *Arabidopsis*, respectively (Supplementary Figure 3). Similar conserved domain architecture (CDART) analysis with the NCBI database showed that the conserved domain of OsLMP1 exhibits the highest homology with the ubiquitin-specific protease UBP8 (*Saccharomyces cerevisiae*) (Supplementary Figure 4).

**FIGURE 4:**
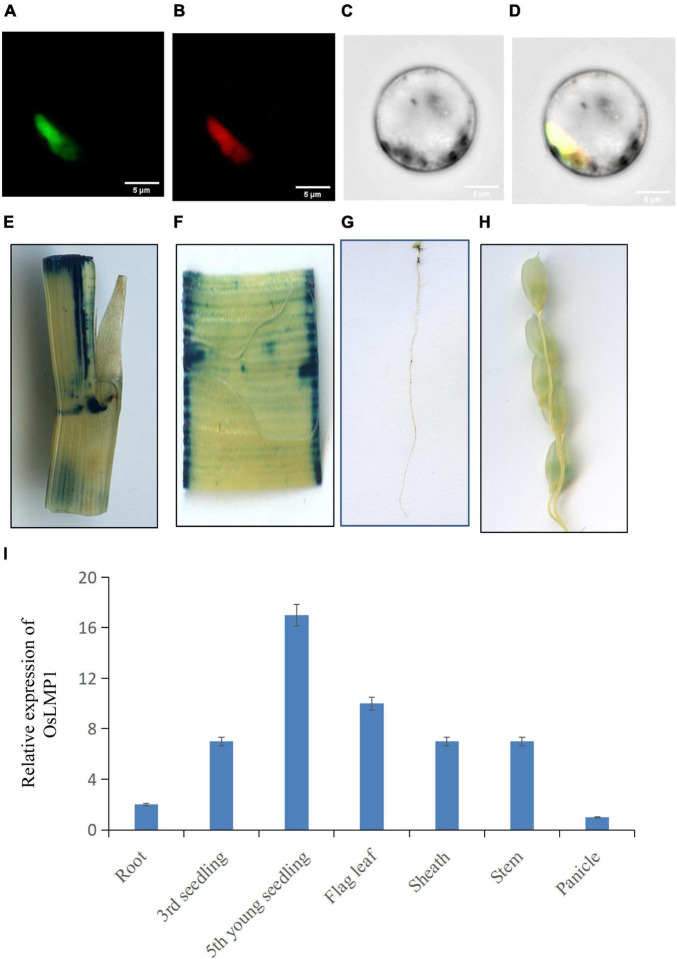
Subcellular location and the expression pattern of OsLMP1. **(A–D)** The 35S:OsLMP1:GFP vector was transformed into rice protoplasts, and the green fluorescent signal was visualized using confocal microscopy. OsMADS3, which localizes to the nuclei, was selected as the marker. The results revealed colocalization of the GFP signal with OsMADS3 fluorescence, indicating that OsLMP1 is mainly targeted to the rice nucleus. Bar: 100 μm. **(E–H)** Transgenic plants containing the OsLMP1-1391Z vector were stained using GUS dye at the flowering stage. **(E)** Stem; **(F)** leaf blade; **(G)** root; **(H)** panicle. The results showed that the stem, leaf blade and root were more deeply stained, whereas the panicle was weakly stained. **(I)** qRT-PCR analysis of OsLMP1 in the root, seedling, flag leaf, stem, sheath, and panicle tissues of the wt. The results showed that OsLMP1 is mainly expressed in young leaf at the early tiller stage.

OsLMP1 encodes a deubiquitinase composed of 1,055 amino acids with a molecular weight of 116 kDa. To assess the localization pattern of OsLMP1, a fusion construct (35S:OsLMP1:GFP) and pMcherry (35S:OsMADS3:RFP, as a control) were transiently transformed into rice protoplasts by PEG transformation method. After 16 h of incubation at 28°C in the dark, the transformed protoplasts were observed using confocal laser scanning microscopy. GFP fluorescence in the image almost merged with the pMcherry fluorescence. Thus, the localization assay showed that OsLMP1 is mainly localized in the nucleus (Figures 4A–D).

The 2K promoter of *OsLMP1* was amplified and fused with *p*Cambia1391Z. The fused recombinant construct (OsLMP1-1391Z) was transformed into *Nipponbare* calli. A GUS staining experiment in the positive lines showed that *OsLMP1* is preferably expressed in the leaf, stem and root and weakly expressed in the panicle (Figures 4E–H). The qRT-PCR results showed that OsLMP1 is most highly expressed in the young seedlings (at the tiller stage); moderately expressed in flag leaves, leaf blades; and weakly expressed in the roots and panicles, which is generally consistent with the GUS staining results (Figure 4I).

### Transcriptome Analysis

**FIGURE 5:**
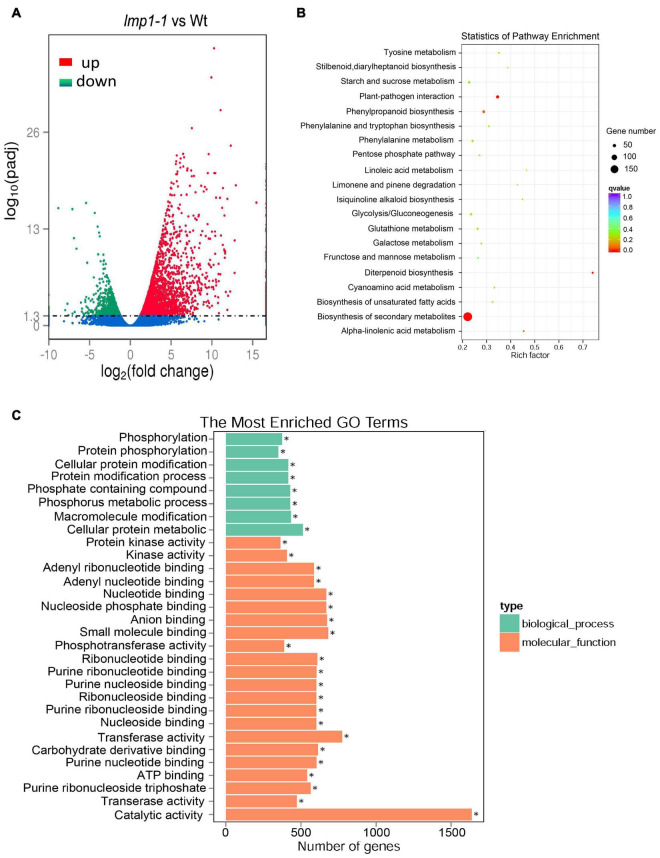
Transcriptome analysis and comparison of the *lmp1-1* and wt lines. **(A)** The *X*-axis indicates the multiple of the difference after log2 conversion, and the *Y*-axis indicates the significance value after –log10 conversion for the *lmp1-1* and wt lines. Points represent the percentage of the number of DEGs (ratios of the number of DEGs to the total number of detected genes enriched in the same GO terms) identified by paired transcriptome analysis. The numbers of upregulated DEGs are on the right of the backslashes, and the numbers of downregulated DEGs are on the left. vs., versus. **(B)** KEGG enrichment analysis of the *lmp1-1* and wt liens. The *X*-axis indicates the enrichment ratio (the ratio of the number of genes annotated to an entry in the selected gene set to the total number of genes annotated to the entry in the species). The *Y*-axis indicates the KEGG pathway, and the size of the bubble indicates the number of genes. The color represents the enriched *Q*-value; the darker the color is, the smaller the *Q*-value is. **(C)** GO annotation of the DEGs from the leaves of the *lmp1-1* and wt lines at the later tiller stage. The top 30 GO terms with *P* < 0.05 are shown here, and more information about the GO annotations is shown in Supplementary Table 2.

To determine the regulatory pathways that *OsLMP1* may be involved in, transcriptome analysis was conducted using wild-type and *lmp1-1* mutant plants at the later tiller stage. A total of 2,737 genes were upregulated, and 1,064 genes were downregulated in *lmp1-1* (at least three repeats, *p* ≤ 0.01) (Figure 5A and Supplementary Table 1). Some disease resistance-responsive family proteins, such as WRKYs and PRs, were activated in the *lmp1-1* plants, which was consistent with the qRT-PCR results. We then performed Gene Ontology (GO) analysis to classify and identify the functions of the differentially expressed genes (DEGs) in the *lmp1-1* plants (Figure 5C). A total of 64 GO terms were divided on the up-regulated datasets (at least three repeats, *p* ≤ 0.01). Among the GO terms 20 GO terms belong to biosynthetic process 42 terms belong to molecular function, and 2 terms belong to Component process (Supplementary Table 2). The “molecular function,” “biosynthetic process,” and “catalytic activity” terms were the terms most enriched in the DEGs, with *p*-values of 1.97E-10, 0.003708, and 6.78E-19, respectively (upregulated) (Supplementary Figure 7). A total of 38 GO terms were enriched in the downregulated DEGs, among which “catalytic activity,” “ion binding,” and “transferase activity” were the most enriched in the downregulated DEGs, with *p*-values of 5.01E-05, 0.0042861, and 6.72E-08, respectively (downregulated) (Supplementary Figure 8). Among the GO terms enriched in the downregulated and upregulated DEGs, the “catalytic activity” term was among the top three, indicating that OsLMP1 may regulate plant disease resistance through its catalytic activity. The biological pathways enriched in the *lmp1-1* mutant plants were also explored, and we performed KEGG enrichment analysis of the DEGs between the *lmp1-1* mutant and wild-type plants. The DEGs were mainly enriched in the “plant–pathogen interaction,” “phenylpropanoid biosynthesis,” and “biosynthesis of secondary metabolites” pathways (Figure 5B). In-depth analysis suggested that the upregulated DEGs were significantly enriched in seven pathways (*P* ≤ 0.01), among which (Supplementary Table 3 and Supplementary Figure 5) the “plant–pathogen interaction,” “alpha-linolenic acid metabolism,” “diterpenoid biosynthesis,” “phenylpropanoid biosynthesis,” “biosynthesis of secondary metabolites,” “biosynthesis of unsaturated fatty acids,” and “phenylalanine, tyrosine and tryptophan biosynthesis” pathways were the most enriched and prominent (upregulated). In contrast, the downregulated DEGs were enriched in six predominant pathways, among which the “diterpenoid biosynthesis,” “carbon fixation in photosynthetic organisms,” “biosynthesis of secondary metabolites,” “cyanoamino acid metabolism,” “carotenoid biosynthesis,” and “photosynthesis” pathways were the most significantly enriched pathways, with *p*-values of 0.00021, 0.00333, 0.00472, 0.01487, 0.01559, and 0.02610, respectively (downregulated) (Supplementary Table 3 and Supplementary Figure 6). These results indicated that *OsLMP1-*mediated disease resistance is likely associated with phenylalanine metabolic pathway in up-regulated database sets and the photosynthesis in down-regulated database sets.

### *OsLMP1* Encodes a Functional Deubiquitinase Enzyme

**FIGURE 6:**
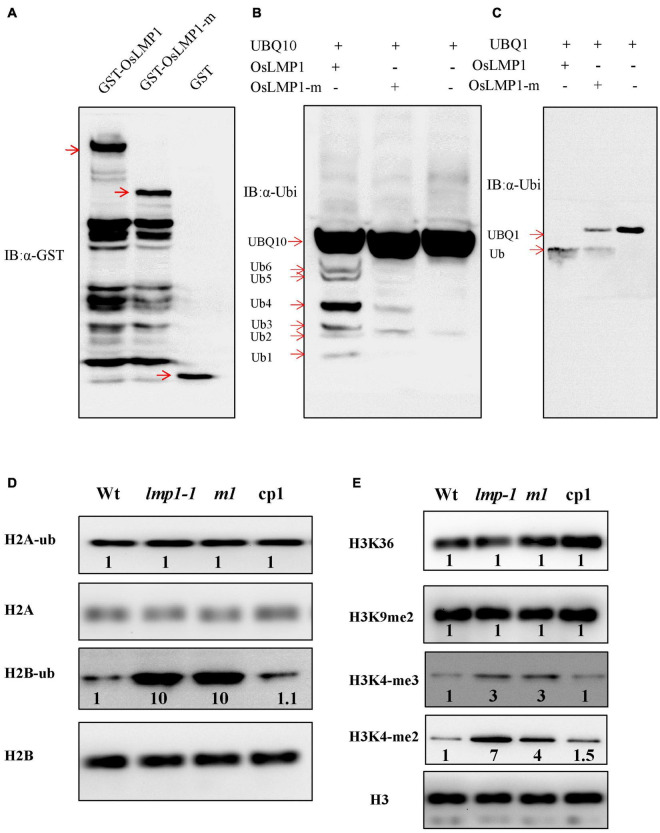
OsLMP1 is an active deubiquitinase. **(A)** The fused proteins GST-OsLMP1, GST-OsLMP1-m, and GST were purified in *E. coli*. Western blotting was conducted using an anti-GST antibody. Red arrows denote the expected bands. **(B)** The fused proteins GST-OsLMP1, GST-OsLMP1-m, and GST were coexpressed with His-UBQ10 in *E. coli*. Western blotting was conducted using an anti-ubiquitin (α-ubi) antibody. Immunoblot analysis showed that GST-OsLMP1 could cleave His-UBQ10, but GST-OsLMP1-m did not cleave His-UBQ10. Red arrows denoted the ubiquitination precursor bands. **(C)** The fused proteins GST-OsLMP1, GST-OsLMP1-m, and GST were coexpressed with His-UBQ1 in *E. coli*. Western blotting was conducted using an anti-ubiquitin antibody (α-ubi). Immunoblot analysis showed that GST-OsLMP1 could cleave His-UBQ1, but GST-OsLMP1-m did not cleave His-UBQ1. Red arrows denoted the ubiquitination precursor bands. **(D)** Western blot analysis of purified rice histone proteins at the later tiller stage with an anti-H_2_A/H_2_B antibody and anti-ubiquitin antibody. The levels of H_2_B-ub were higher in *lmp1-1* and crisp-m1 plants than in wt plants. Band intensities were quantified using ImageJ analysis of the Western blots. The fold change shown above the blot is relative to wt controls normalized by antibody against H_2_A or H_2_B in each lane (*n* = 3). **(E)** Western blot analysis of purified rice histone proteins at the later tiller stage was conducted with an anti-H_3_ antibody or specific antibodies. The results show that the levels of H_3_K_4_-me2 and H_3_K_4_-me3 were higher in *lmp1-1* and crisp-m1 plants than in wt plants. Band intensities were quantified using ImageJ analysis of the Western blots. The fold change shown above the blot is relative to wt controls normalized by anti-H_3_ antibody in each lane (*n* = 3).

Since *OsLMP1* contains a C-terminal hydrolase catalytic domain, we determined whether OsLMP1 has deubiquitinase enzyme activity. Vectors for GST-OsLMP1, GST-OsLMP1-m (in which an “A” base was inserted at bp 2,604, generating a truncated protein) and GST (as a control) were constructed and expressed in *E. coli*. Western blot experiments using an antibody against GST showed that purified GST-OsLMP1 showed the expected molecular weight of 142 kDa and that GST-OsLMP1-m showed a molecular weight of 94 kDa, as expected for truncation (Figure 6A). Then, the GST-OsLMP1 and GST-OsLMP1-m vectors were coexpressed with the substrate His-UBQ10 in *E. coli*, respectively. Western blot assays were conducted using an anti-ubiquitin antibody. The results showed that GST-OsLMP1 could cleave ubiquitin molecules from the substrate His-UBQ10, but the ubiquitin precursor His-OsUBQ10 was not cleaved by GST-OsLMP1-m (Figure 6B). Similarly, the substrate His-OsUBQ1 was completely cleaved by GST-OsLMP1, but only a small amount was cleaved by GST-OsLMP1-m (Figure 6C). The above results indicated that OsLMP1 has functional deubiquitinase activity, and the mutant OsLMP1 could not cleave ubiquitin molecules or had weak ubiquitin cleavage activity.

### OsLMP1 Deubiquitinates H_2_B

Similar CDART analysis showed that the conserved domains of OsLMP1 show the highest homology with the ubiquitin-specific protease UBP8 from *S. cerevisiae* (Supplementary Figure 3). In *S. cerevisiae*, Ubp8, Sgf11, Sus1, and Sgf73 form a large subcomplex known as the deubiquitination (DUB) module (Morgan et al., 2016). The catalytic domain of Ubp8 contacts H_2_B, leading to H_2_B deubiquitination at multiple stages of nucleosome disassembly and reassembly during transcription (Morgan et al., 2016). Thus, we speculated that OsLMP1 may also deubiquitinate histone H_2_A or H_2_B. Monoclonal antibodies specific for ubiquitinated histone H_2_A or H_2_B were used to detect the levels of H_2_B-ub and H_2_A-ub in the wild-type, *lmp1-1*, crisp-m1 and complementation lines at the later tiller stage. The results showed that the amount of H_2_A-ub in the mutants was comparable to that in the wild-type lines, but the amount of H_2_B-ub was significantly greater (by ∼10 times) in the *lmp1-1* and crisp-m1 lines compared to the wild-type line (Figure 6D). The amount of H_2_B-ub in the complementation lines was restored to the level in the wild-type line. The above experimental results indicated that *OsLMP1* may function by deubiquitinating H_2_B.

H_2_B ubiquitination is often a prerequisite for H_3_K_4_ and H_3_K_9_ methylation, and the two processes are closely coupled (Sridhar et al., 2007). Western blot assays at the later tiller stage were used to detect the H_3_ methylation status in the wild-type, *lmp1-1*, crisp-m1 and complementation lines with an anti-methylated H_3_ antibody (H_3_K_4_me2, H_3_K_4_me3, H_3_K_9_me2, and H_3_K_36_me2). The band intensities were quantified using ImageJ analysis of western blots. The fold change shown above the blot is relative to wt controls normalized by antibody against H_2_A or H_2_B in each lane. The imaging results showed that the levels of H_3_K_4_me2 and H_3_K_4_me3 methylation were significantly increased by at least 3 times in the *lmp1-1* and crisp-m1 lines compared with the wild-type line, whereas H_3_K_9_me2 and H_3_K_36_me2 methylation levels in the *lmp1-1* and crisp-m1 lines were comparable to those in the wild-type line (Figure 6E). The H_3_K_4_me2 and H_3_K_4_me3 methylation levels in the complementation lines were restored to the level in the wild-type line. Dot1L and Set1 build a ‘histone crosstalk’ bridge between ubiquitination of histone H2B on K120 and histone H3 (Worden et al., 2019). Our experiment supported that methylation of histone H_3_K_4_ was associated with active transcription, which was closely related to H_2_B deubiquitination.

### *OsLMP1* Is Involved in the Deubiquitination of *OsPAL6* and *OsPAL7* Chromatin

**FIGURE 7:**
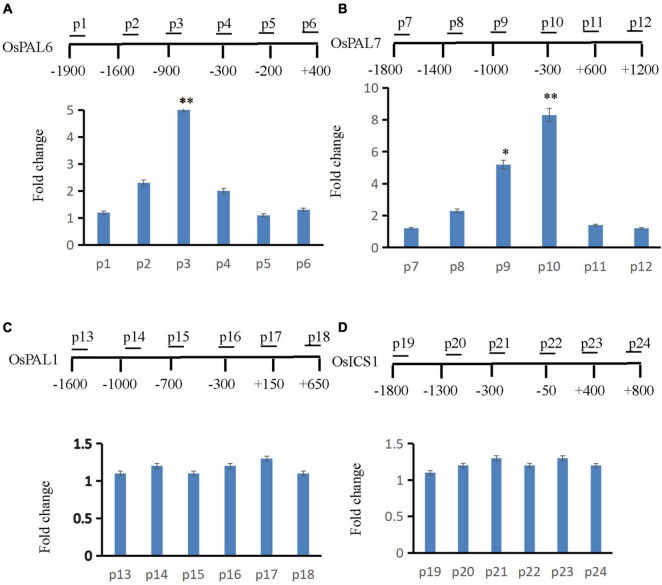
ChIP assay to analyze SA synthesis pathway genes. The supernatant was immunoprecipitated with an anti-H_2_B-Lys 120 antibody, and the resulting DNA of wt was eluted and subjected to quantitative PCR to assess genes involved in the SA synthesis pathway (**A:** OsPAL6, **B:** OsPAL7, **C:** OsPAL1, and **D:** OsICS1). The immunoprecipitated genomic fragments were quantified using real-time quantitative PCR and normalized to OsActin1 as an internal control.

Gene expression is often coupled with increased H_2_B monoubiquitination (Batta et al., 2011). Thus, *OsLMP1* may deubiquitylate H_2_B at the chromatin level. Many SA synthesis genes were activated in the *lmp1-1* line (Supplementary Table 5). The expression levels of those SA synthesis genes were verified by qRT-PCR (Supplementary Figure 9), and the results were essentially consistent with the transcriptome data. In addition, the SA content in the *lmp1-1* line at the heading stage was measured. The results showed that the SA content was significantly higher in the *lmp1-1* line than in the wild-type line (Figure 1M). Because SA accumulated in the mutant plants, we speculated that the SA synthesis pathway genes may had undergone ubiquitination modification and thus the immune responses were activated in mutants. We examined this hypothesis using chromatin immunoprecipitation qPCR (ChIP -qPCR). Transcriptome analysis showed that OsLMP1 mediates immune responses associated with the phenylalanine metabolic pathway (Supplementary Table 5). SA synthesis pathway genes (*OsPAL1, OsPAL6, OsPAL7*, and *OsICS1*) were selected for ChIP -qPCR analysis. ChIP-qPCR assays at the 5th leaf stage revealed substantial levels of hyperubiquitinated H_2_B in *OsPAL6* and *OsPAL7* chromatin (Figures 7A,B), but not in *OsPAL1* or *OsICS1* chromatin in wild type (Figures 7C,D). The hyperubiquitinated region was located near the translation initiation codon in *OsPAL6* and *OsPAL7*. The degree of ubiquitination ranged from ∼5 to 8 times higher than ubiquitination of the wild-type *OsPAL6* and *OsPAL7* chromatin (Figures 7A,B). The effect on *OsPAL7* monoubiquitination was more significant than that on *OsPAL6* monoubiquitination. In all, examination of H_2_B-ub throughout the length of OsPALs chromatin by chromatin immunoprecipitation (ChIP) analysis revealed enrichment of this histone modification within the promoter of the genes in wild type (Figures 7A,B).

## Discussion

### OsLMP1 Regulates the Immune Response in Rice

Lesion mimic mutants are ideal materials for the study of disease resistance. At present, at least 31 genes have been cloned in rice (Supplementary Table 4), and most act at the transcriptional level. Epigenetic modification is a posttranscriptional regulatory mechanism and plays an important role in the rice immune response. The JMJ705 protein, which contains the Jumonji C domain, is involved in the defense response in rice as it modifies histone H_3_ lysine 27 trimethylation (Li et al., 2013). PigmR confers broad spectrum resistance and is subjected to tight epigenetic regulation (Deng et al., 2017). In this study, we cloned *OsLMP1* by map-based cloning and found that it encodes a deubiquitinase enzyme. The functions of UBP family members in plants have rarely been reported for the following reasons. First, many UCH family members exhibit functional redundancy in plants. For example, AtUBP3 and AtUBP4 are essential for pollen development and transmission in *Arabidopsis* (Doelling et al., 2007). GIGANTEA recruits the deubiquitylases AtUBP12 and AtUBP13 to regulate accumulation of the ZTL photoreceptor complex (Lee et al., 2019). Thus, it is difficult to identify the function in a single gene. However, sequence analysis showed that the rice genome contains no proteins with an amino acid similarity with *OsLMP1* of more than 20%, suggesting that rice contains no redundant genes; thus, a single mutation in *OsLMP1* causes a lesion mimic phenotype.

A series of assays confirmed that OsLMP1 regulates the plant immune response through the following findings. First, in the *lmp1-1* mutant, the expression of pathogenesis-related genes, such as OsWRKY42, OsPR1, and PBZ1, was greatly increased compared to that in the wild-type line, which indicates that the defense response in the *lmp1-1* mutant is significantly activated. Second, DAB staining and H_2_O_2_ measurements experiments pointed out that H_2_O_2_ contents were greatly increased in the mutant leaves, which indicated that PCD was activated and thus resulted in resistance to bacterial blight. Third, the amount of H_2_B-ub was significantly increased by ∼10 times in the *lmp1-1* mutant. *OsLMP1* functions by deubiquitinating H_2_B. Fourth, the SA content was higher in the *lmp1-1* mutant than in the wild-type line. A ChIP-qPCR assay indicated that OsLMP1 was more enriched in the promoters of *OsPAL6* and *OsPAL7* than other *OsPALs* family members. The above four lines of evidence verified that OsLMP1 regulates the immune response by histone ubiquitination.

### OsLMP1 Mediates Histone Ubiquitination in Rice

The ubiquitination and deubiquitination of histone H_2_B, like other histone modifications, regulate chromosome structure and gene transcription (Hu, 2012). Catalytic histone ubiquitination and deubiquitination enzymes have been successfully identified and cloned in yeast, animals and plants. Usp22 regulates histone H_2_B monoubiquitination and exhibits both oncogenic and tumor-suppressor roles in cancer (Jeusset and McManus, 2017). The deubiquitinase OTLD1 targets histone H_2_B to regulate seed size in *Arabidopsis* (Keren and Citovsky, 2016). However, only a few deubiquitinases have been identified in rice. OTUB1, due to its deubiquitinase activity, defines a new plant type associated with higher grain yield (Wang et al., 2017b). OsUBP15, a deubiquitinase, plays an important role in regulating grain width and size (Shi et al., 2019). Recent research has shown that the posttranslational regulation of H_2_B participates in non-host resistance and pathogen defense in plants (Ramirez-Prado et al., 2018). For example, AtHUB1 and AtHUB2, which encode two RING E3 ubiquitin ligase enzymes, catalyze H_2_B monoubiquitination. AtHUB1 and AtHUB2 are essential to fungal pathogens in *Arabidopsis* (Dhawan et al., 2009). Furthermore, reduced tomato H_2_B monoubiquitination by SiHUB1 and SiHUB2 knock down enhanced plant sensitivity to *Botrytis cinerea* (Zhang et al., 2015). In this study, we tested whether OsLMP1 mediates histone ubiquitination in rice. In *E. coli* assays, GST-OsLMP1 could cleave the ubiquitination precursor (Figure 6). Purified GST-OsLMP1 appeared as not only the targeted band but also many unknown bands at small molecular weights (Figure 6A). We speculated that the OsLMP1 protein may undergo unknown modifications, or the observation could have been due to incomplete expression of the full-length reading frame of OsLMP1. In this experiment, we found that the OsLMP1-m lanes contained some bands that were present in the wild-type OsLMP1 sample but not in the negative control (Figure 6B). We speculated that mutant OsLMP1 has weak deubiquitination activity, especially that in the OsLMP1 lane, with OsU BQ1 as a substrate (Figure 6C).

The ChIP-qPCR showed that OsLMP1 may be more enriched in the promoters of *OsPAL6* and *OsPAL7* but not those of *OsPAL* genes. He reported that planthopper resistance was regulated by SA from the PAL pathway in rice (He et al., 2020). In the study, the level of SA is significantly reduced in the Os*PAL*6 co-suppressed or Os*PAL* RNAi plants, but increased in the plants overexpressing *OsPAL8*. This study provide additional support for the notion that the PAL pathway is an important route of SA biosynthesis in rice (He et al., 2020). Higher SA content in *lpm1-1* was perhaps a combined action of OsPAL6 and OsPAL7. The expression levels of *OsPAL2* and *OsPAL4* were slightly increased in the mutants, which may have been due to feedback regulation of SA signaling. Isochorismate synthase (ICS) and phenylalanine ammonia lyase (PAL) are two important genes in the plant SA synthesis pathway. OsPALs, and not OsICS1, may be primarily responsible for SA synthesis in rice (He et al., 2020). However, AtICS1 is mainly responsible for SA synthesis in *Arabidopsis*. Furthermore, the temporal and spatial expression patterns and mechanisms of PALs and ICSs in *Arabidopsis* and rice need to be further studied. In addition, we tried to verify the interaction of OsLMP1 with H_2_B variants, but the results did not verify their interaction. Thus, OsLMP1 and H_2_B may be connected through a new regulatory link.

### A Suggested Model of OsLMP1 Regulation

We suggest a model (Supplementary Figure 11) in which the *OsLMP1* complex maintains deubiquitinase activity under normal growth conditions in the wild-type, which maintains the level of histone H_2_B ubiquitination at the background level or lower at different developmental stages, thus inhibiting the expression of disease resistance-related genes. In the *lmp1-1* mutant, the deubiquitination activity of the OsLMP1 complex is decreased, and the levels of histone H_2_B ubiquitination and methylation are increased, which activates the expression of disease resistance-related genes. Under conditions of pathogen infection, unknown factors may reduce OsLMP1 complex activity (Supplementary Figure 10), increasing H_2_B ubiquitination levels, thus activating the expression of disease resistance-related genes and conferring resistance in plants (Figure 7).

In summary, this study preliminarily reveals that OsLMP1 regulates plant disease resistance by epigenetically modifying histone H_2_B. OsLMP1 loss of function led to high levels of histone H_2_B ubiquitination and histone H_3_-K_4_me2/3 methylation, directly activated SA synthesis genes (*OsPAL6* and *OsPAL7*) and improved plant disease resistance. This pathway may be conserved in animals and plants. However, whether other UCH family members also participate in H_2_B deubiquitination requires further study. Moreover, this study also provides important insight suggesting that other UCH family members in plants induced by pathogenic bacteria are also related to plant disease resistance. In addition, because the deubiquitination and ubiquitination processes are coupled, we would expect to find ubiquitination-related proteins in rice related to disease resistance.

## Data Availability Statement

The datasets presented in this study can be found in online repositories. The names of the repository/repositories and accession number(s) can be found below: https://www.ncbi.nlm.nih.gov/genbank/ and https://www.ncbi.nlm.nih.gov/sra/PRJNA694229.

## Author Contributions

JS and YC performed the map-based cloning of OsLMP1. WS made the expression analysis and subcellular localization experiments. YW and WS performed western blotting and ChIP experiments. YC conducted the transgenic experiments. ZZ and TL designed the research and wrote the manuscript. All authors contributed to the article and approved the submitted version.

## Conflict of Interest

The authors declare that the research was conducted in the absence of any commercial or financial relationships that could be construed as a potential conflict of interest.

## Publisher’s Note

All claims expressed in this article are solely those of the authors and do not necessarily represent those of their affiliated organizations, or those of the publisher, the editors and the reviewers. Any product that may be evaluated in this article, or claim that may be made by its manufacturer, is not guaranteed or endorsed by the publisher.

## Footnotes

1
https://www.ncbi.nlm.nih.gov/sra/PRJNA694229

2
https://bioinfo.ut.ee/primer3-0.4.0/

## Supplementary Material

Supplementary Figure 1 Enhanced resistance in the *lmp1-1* mutant to *Magnaporthe oryzae* and *Xanthomonas oryzae* pv. *oryzae* isolates.

Supplementary Figure 2 The expression level of OsLMP1 in the complementation lines (CP1–CP3) was restored to the expression level in the wild-type line.

Supplementary Figure 3 Phylogenetic tree showing the predicted relationships between OsLMP1 and homologous proteins in rice and *Arabidopsis*.

Supplementary Figure 4 Similar conserved domain architecture (CDART) analysis with NCBI showed that the conserved domain in OsLMP1 exhibits the highest homology with the ubiquitin-specific protease UBP8 (*Saccharomyces cerevisiae*).

Supplementary Figure 5 Up-regulated genes KEGG enrichment analysis of the *lmp1-1* and wt lines.

Supplementary Figure 6 Down-regulated genes KEGG enrichment analysis of the *lmp1-1* and wt lines.

Supplementary Figure 7 GO annotation of the up-regulated DEGs from the leaves of the *lmp1-1* and wt lines at the later tiller stage.

Supplementary Figure 8 GO annotation of the down-regulated DEGs from the leaves of the *lmp1-1* and wt lines at the later tiller stage.

Supplementary Figure 9 The expression levels of SA synthesis genes, including OsPAL1-7 and OsICS1, were verified by qRT-PCR **(A–H)**.

Supplementary Figure 10 Time course of OsLMP1 expression in the later tiller stage in the seedlings of wt plants treated with rice bacterial blight (PXO99) using the leaf cutting method.

Supplementary Figure 11 A model of the role of OsLMP1 in the defense response.

Supplementary Table 1 Differential analysis of the *lmp1-1* vs. wt lines.

Supplementary Table 2 Results of GO enrichment analysis of DEGs in the *lmp1-1* line vs. the wt line.

Supplementary Table 3 Results of KEGG analysis of the *lmp1-1* line vs. the wt line.

Supplementary Table 4 Cloned lesion mimic genes in rice.

Supplementary Table 5 Fold change in the expression of SA-related genes in the *lmp1-1* line vs. the wt line.

Supplementary Table 6 Primer sequences used.
