# Supplementary material for: OsLMP1, Encoding a Deubiquitinase, Regulates the Immune Response in Rice
Source: Front Plant Sci. 2022 Jan 18;12:814465. doi: 10.3389/fpls.2021.814465 (PMC8805587; doi:10.3389/fpls.2021.814465)
Supplement: Supplementary Figure 1 — Enhanced resistance in the lmp1-1 mutant to Magnaporthe oryzae and Xanthomonas oryzae pv. oryzae isolates. [file Data_Sheet_1.docx]

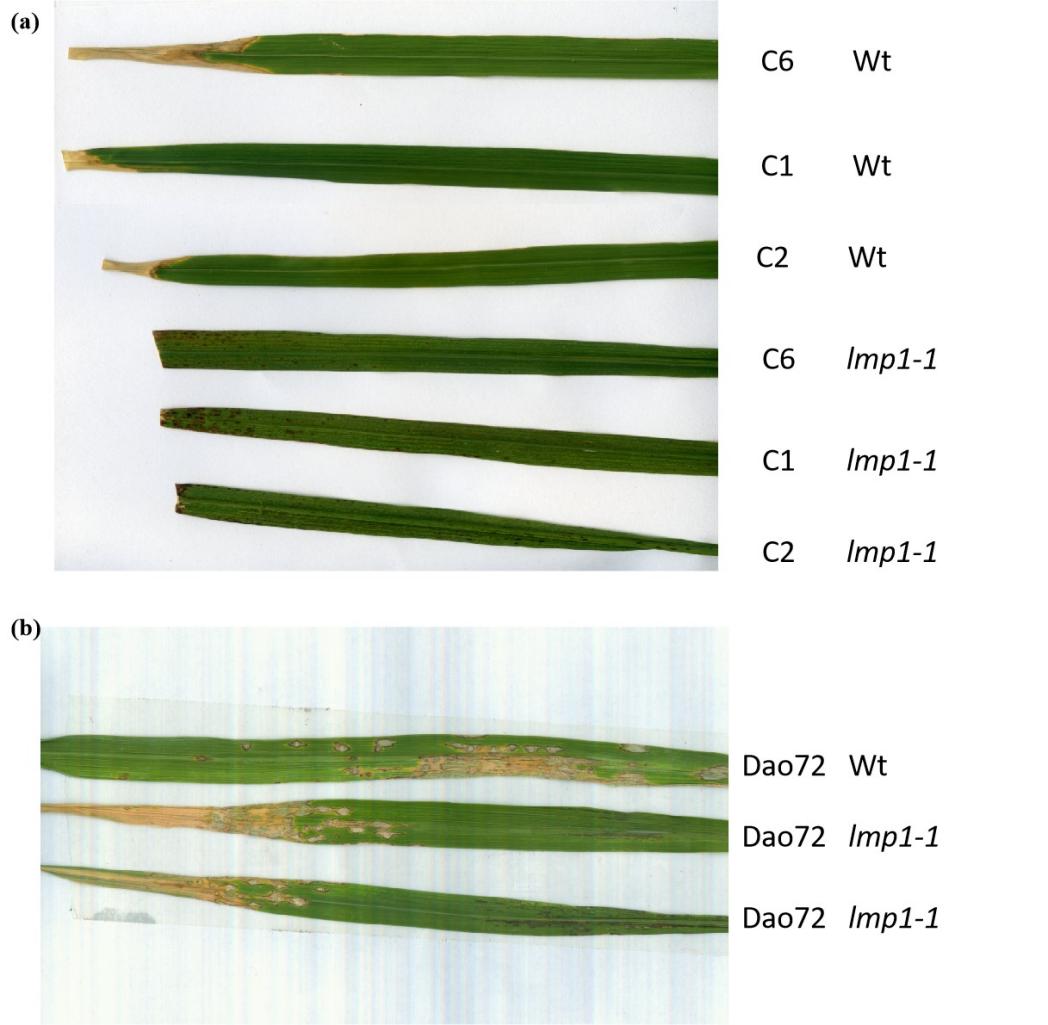


**Fig. S1.** Enhanced resistance in the *lmp1-1* mutant to Magnaporthe oryzae and Xanthomonas oryzae pv. oryzae isolates. (a) Reactions to three Xanthomonas oryzae pv. oryzae isolates (C1,C2,C6). The variety Nipponbare (wt) was used as a control. (b) Reactions to one Magnaporthe oryzae isolate (Dao72). The result showed that some Magnaporthe oryzae and Xanthomonas oryzae pv. oryzae physiologic races showed more enhanced resistance in lmp1-1 than control (wild type) .


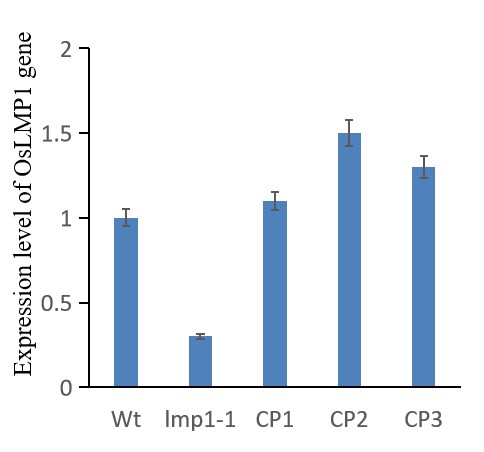


**Fig. S2. The expression level of OsLMP1 in the complementation lines (CP1-CP3) was restored to the expression level in the wild-type line.**


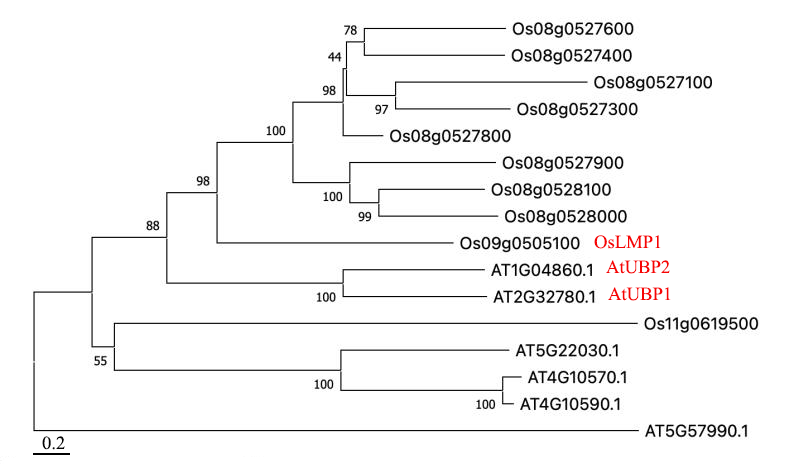


**Fig. S3. Phylogenetic tree showing the predicted relationships between OsLMP1 and homologous proteins in rice and *Arabidopsis*.**

Full-length amino acid sequences of each protein were aligned using CLUSTALW and revised manually. The tree was constructed using the neighbor-joining method. Neighbor-joining phylogenetic tree analysis based on full-length protein sequences from the OsLMP1 homologous members. The bar represents genetic distance in the phylogenetic tree.


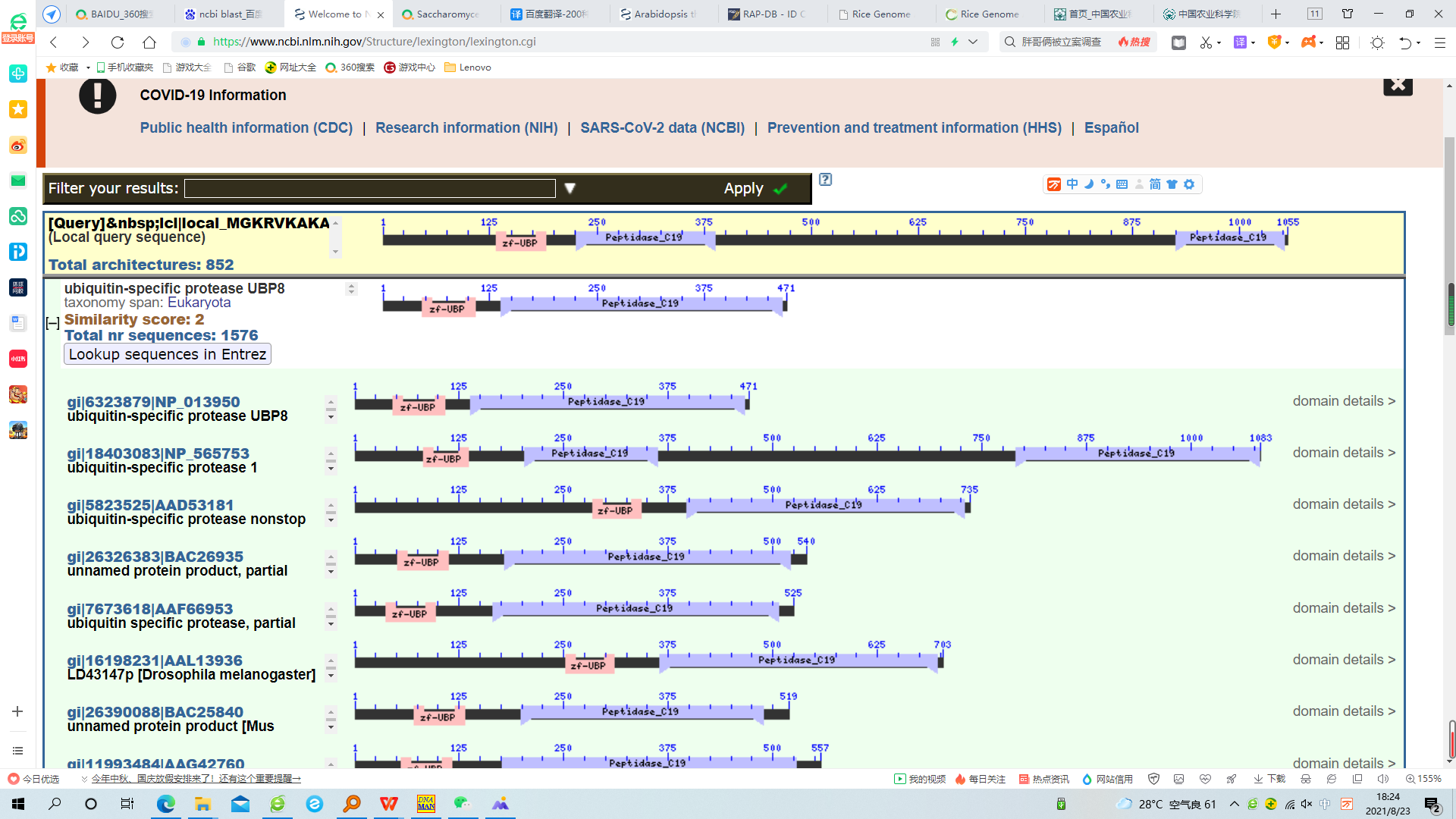


**Fig. S4. Similar conserved domain architecture (CDART) analysis with NCBI showed that the conserved domain in OsLMP1 exhibits the highest homology with the ubiquitin-specific protease UBP8 (*Saccharomyces cerevisiae*).**

**
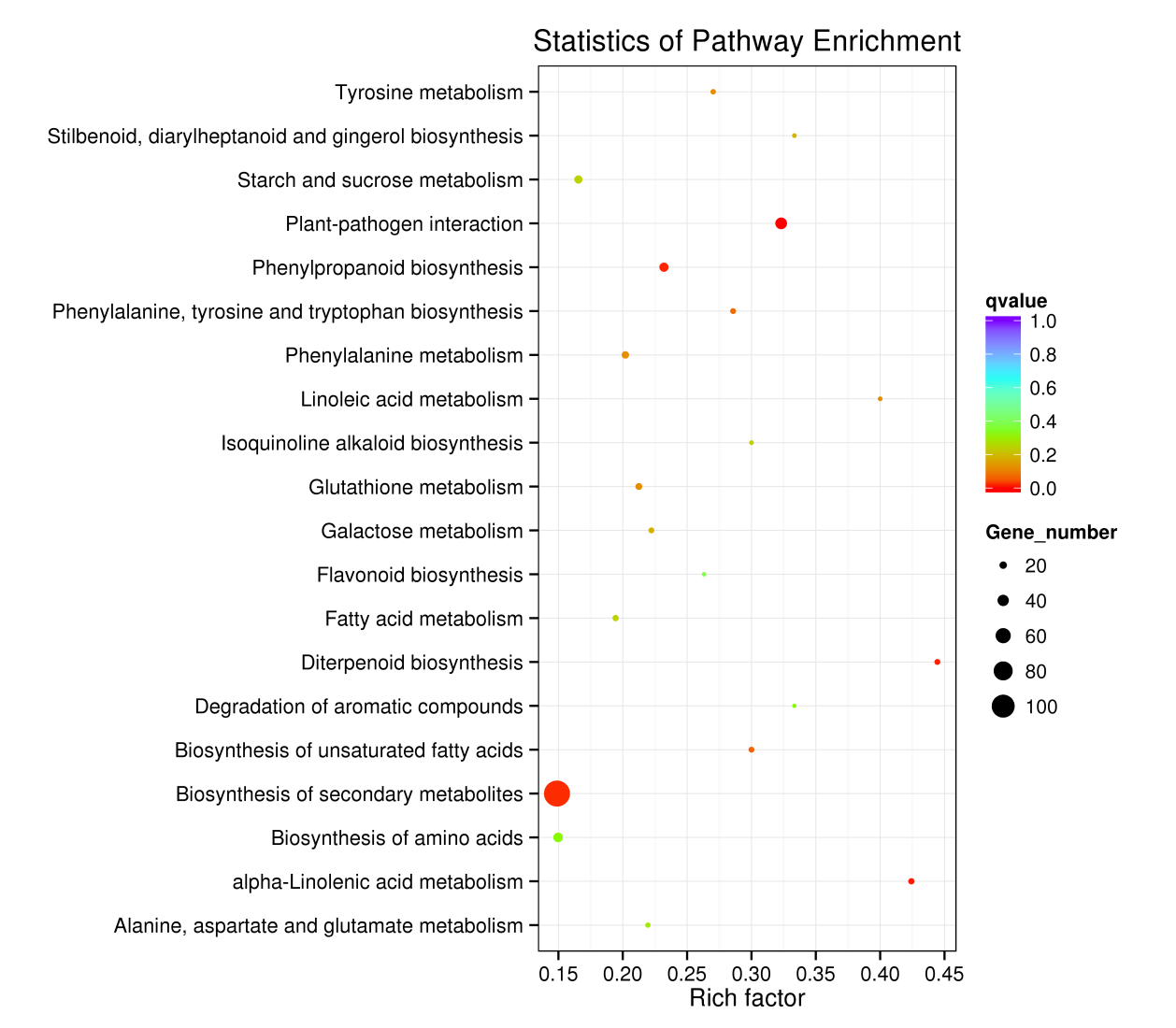
**

**Fig. S5. Up-regulated genes KEGG enrichment analysis of the *lmp1-1* and wt lines.** The X-axis indicates the enrichment ratio (the ratio of the number of genes annotated to an entry in the selected up-regulated gene set to the total number of genes annotated to the entry in the species). The Y-axis indicates the KEGG pathway, and the size of the bubble indicates the number of genes. The color represents the enriched Q-value; the darker the color is, the smaller the Q value is.

**
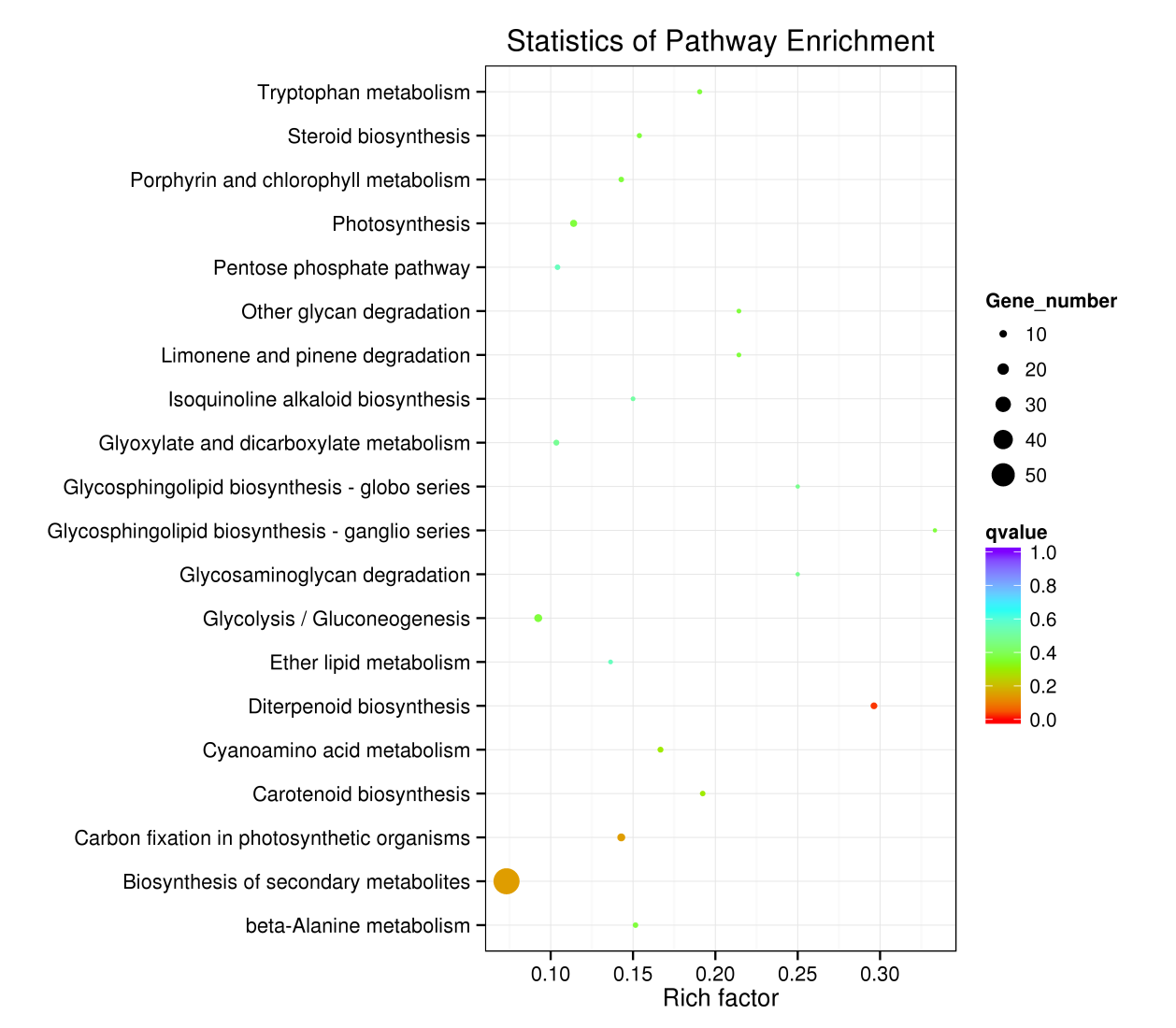
**

**Fig. S6. Down-regulated genes KEGG enrichment analysis of the *lmp1-1* and wt lines.** The X-axis indicates the enrichment ratio (the ratio of the number of genes annotated to an entry in the selected down-regulated gene set to the total number of genes annotated to the entry in the species). The Y-axis indicates the KEGG pathway, and the size of the bubble indicates the number of genes. The color represents the enriched Q-value; the darker the color is, the smaller the Q value is.

**
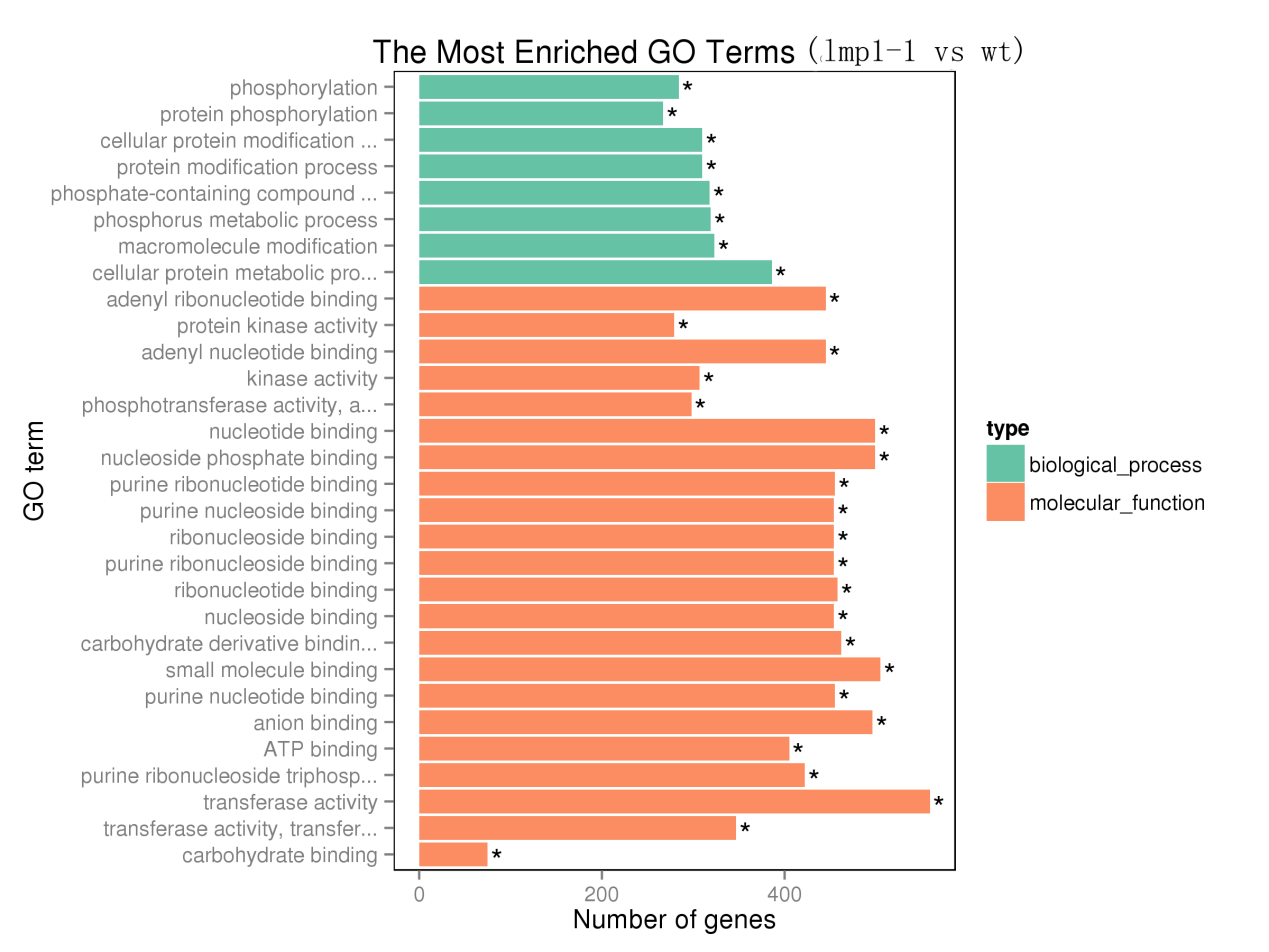
**

**Fig. S7. GO annotation of the up-regulated DEGs from the leaves of the *lmp1-1* and wt lines at the later tiller stage.**

**
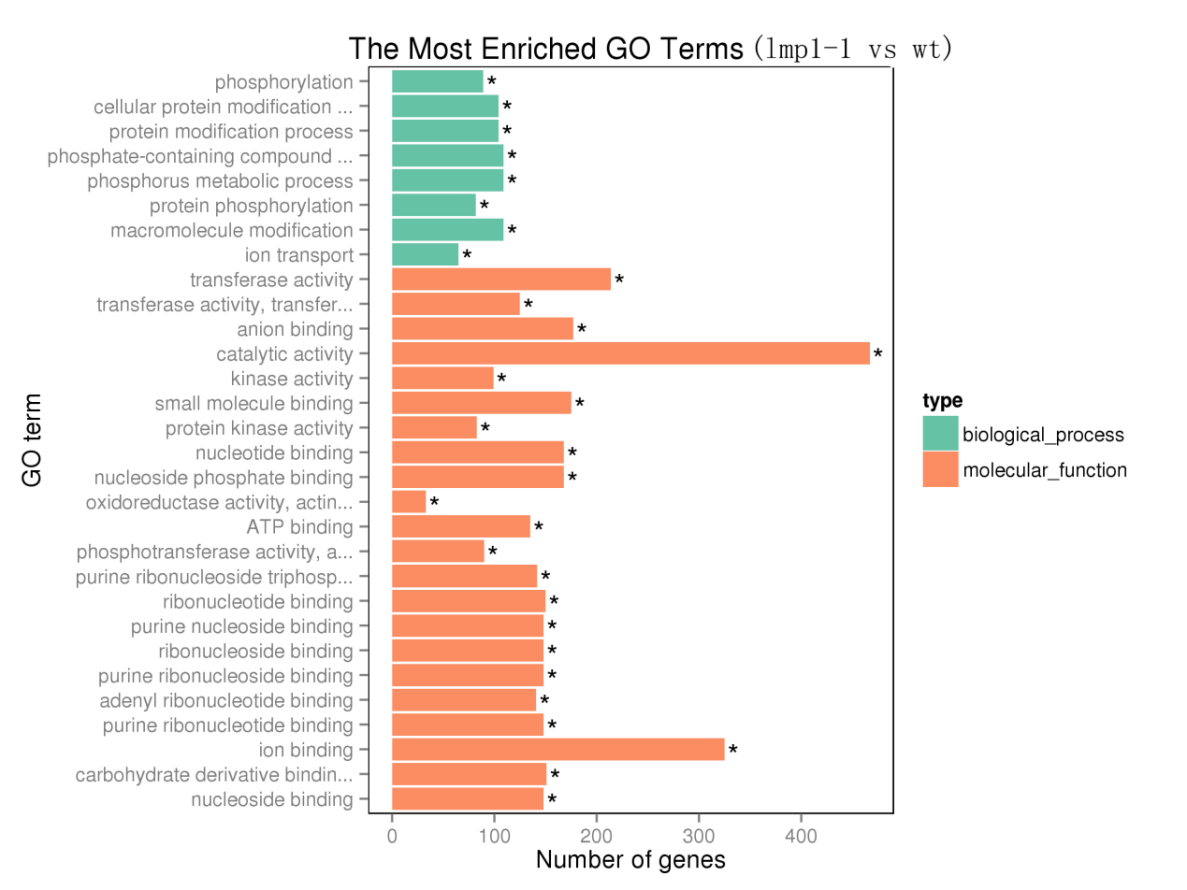
**

**Fig. S8. GO annotation of the down-regulated DEGs from the leaves of the lmp1-1 and wt lines at the later tiller stage.**

**Fig. S9. The expression levels of SA synthesis genes, including OsPAL1-7 and OsICS1, were verified by qRT-PCR (a-h).**


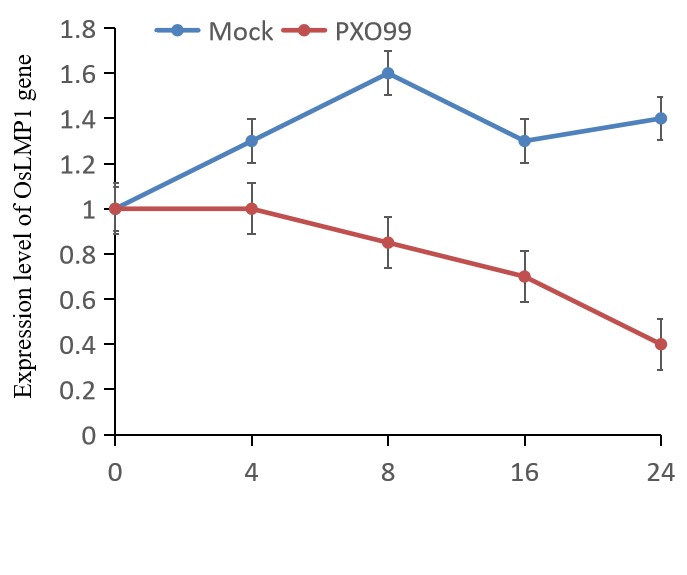


**Fig. S10. Time course of OsLMP1 expression in the later tiller stage in the seedlings of wt plants treated with rice bacterial blight (PXO99) using the leaf cutting method.**

Take samples (the sampling site is 2cm away from the incision) every 4 hours. The result showed that the expression of OsLMP1 after PXO99 treatment decreased significantly. Mock was selected as control (untreated)（n=3）.


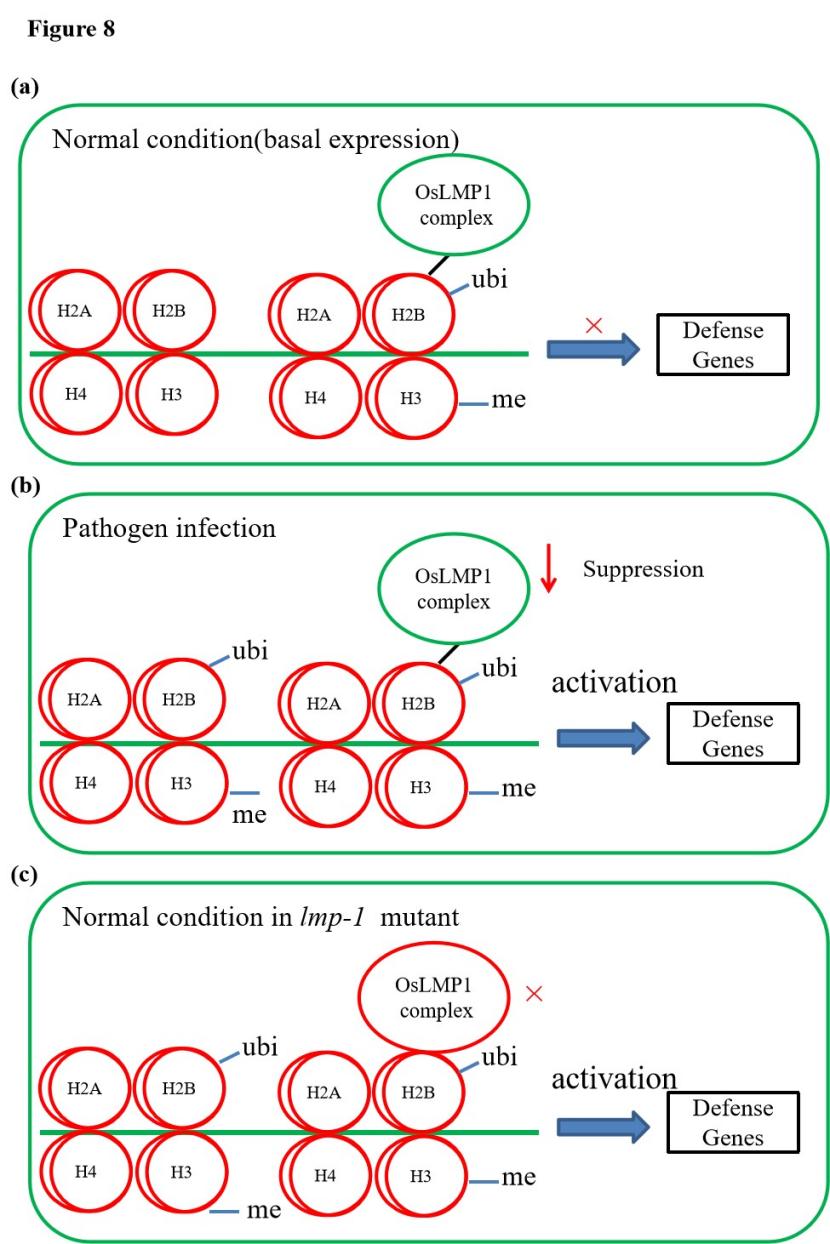


**Fig. S11. A model of the role of OsLMP1 in the defense response.**

In this model, the OsLMP1 complex maintains its deubiquitination activity under normal growth conditions in the wt plants, and the level of H_2_B ubiquitination is maintained at the background level or lower, thus inhibiting the expression of disease resistance-related genes. In *lmp1-1*, the deubiquitination activity of the OsLMP1 complex is decreased, and the ubiquitination and methylation of H_2_B are increased, which activates the expression of disease resistance-related genes. Under pathogen infection conditions, unknown factors reduce *OsLMP1* complex activity and increase H_2_B ubiquitination levels, thus activating the expression of disease resistance-related genes and improving plant resistance.
