# Supplementary material for: OsLMP1, Encoding a Deubiquitinase, Regulates the Immune Response in Rice
Source: Front Plant Sci. 2022 Jan 18;12:814465. doi: 10.3389/fpls.2021.814465 (PMC8805587; doi:10.3389/fpls.2021.814465)
Supplement: Supplementary Table 6 — Primer sequences used. [file Table_6.doc]

**Table S6. Primer sequences were used as followed.**

| **Primers** | **Sequence（5'-3'）** | | | |
| --- | --- | --- | --- | --- |
| **For Mapping** | | | | |
| Indel-1-F/R | TGGGAAGGGCTCCTTTTTAC | | TTCGTCTTGTCGCAATTTGT | |
| Indel-2-F/R | AAGTGCTCGACTCCGATTGT | | ACTGCTGCTGCAGGTTGG | |
| 3017-F/R(SNP) | CACTGTAGTGCTGGCCTTGA | | CCAAACCTGACACCTTCGTT | |
| 3020-F/R(SNP) | GCCCATGGACATCTGAGTTT | | GGCAGAAAGCCGATGTAAGA | |
| **For Mutant Sequencing** | | | | |
| 1-F/R | CCATCCAGCAACATCAATCAGTC | | GCAACAAGAGATGTGCTAAACCA | |
| 2-F/R | GCCAGTCCAAAAGATTGTAAAAGA | | CCAACTGGACTCAATCAAAGAAAC | |
| 3-F/R | ATTCGCATTTGCTACTAAGAGGTC | | ATGCCTTTTCTTTGGGACATCTAT | |
| 4-F/R | GAACAGGCAGACAGCAAAGCAT | | TGGTGGAGTTGAATACGATAATGC | |
| 5-F/R | AACACTCTGGTGAAAACCCTATTG | | TTCATTCTTATTGGTTGTGCTGC | |
| 6-F/R | CTCCGCTGTTTTCTTGATGGTC | | CGGTTCAGTAAACAAAGCCAAG | |
| 7-F/R | GAATCCGAGGAAAGCACAGC | | GTGATGACACACTCTTGGCTGG | |
| 8-F/R | TCCCCTTCATCTCCCCATCT | | ACCTCCTTTCTTCTTCTGCTGC | |
| 9-F/R | GAAGTCAGCAGTGTAGTAGGTGGC | | TCAAGACTAAGCAAGAAACCAAGC | |
| **For OsLMP1 Subcellular Localization** | | | | |
| OsLMP1-GFP-F/R | GGAGCTAGCTCTAGAATGGGGAAGAGGGTGAAGG | | CATGGATCCCCCGGGGTCTCCCACCCTTTCGTA | |
| **For Complementation Construct** | | | | |
| OsLMP1--com-F/R | GTAGAAGAGGTACCCGGGATGGGGAAGAGGGTGAAGG | | GTAGAAGAGGTACCCGGGATGGGGAAGAGGGTGAAGG | |
| **Q-PCR analysis for Pathogen related genes** | | | | |
| OsPR1b-F/R | AACCTTGGCGAGAACCTCTT | | | GCCGGCTTATAGTTGCATGT |
| PR1-F/R | GTCGGAGAAGCAGTGGTACG | | | GGCGAGTAGTTGCAGGTGAT |
| PBZ1-F/R | GGTGTGGGAAGCACATACAA | | | GCTCGTACTCCACCTTGAGC |
| OsCDC48-F/R | ACCGGAAACTTGTTTGATGC | | | CCGTATCAGGTGCAACAATG |
| OsWRKY42-F/R | CACCAATGCAGTCTGCTTCA | | | TAGTACCCGCGTGGATAAGG |
| OsNPR1 | TGAAAGAAGGGACCCACAAC | | | AGGTGGATTTGCACCAGAAC |
| Primers for knocking out the OsLMP1 | | | | |
| OsLMP1-B1’ | | TTCAGAggtctcTctcgACTAGTGGAATCGGCAGCAAAGG | | |
| OsLMP1-gRT1 | | GCATCATCCTGTGGAAGAAAgttttagagctagaaat | | |
| OsLMP1-OsU3T1 | | TTTCTTCCACAGGATGATGCgccacggatcatctgc | | |
| OsLMP1-BL | | AGCGTGggtctcGaccgACGCGTCCATCCACTCCAAGCTC | | |
| GUS staining of the OsLMP1 | | | | |
| OsLMP1-GUS-F | CTGCAGGTCGACGGATCCCAAATCGGTCATTCTTGTCCAC | | | |
| OsLMP1-GUS-R | AAATTTACCCTCAGATCTACCATCGCTTCCGATGCCGAGA | | | |
| Enzyme detection of *OsLMP1* | | | | |
| recombinant GST-OsLMP1 F/R or GST-OsLMP1-m F/R | AAGTTCTGTTCCAGGGGCCCCATATGATGGGGAAGAGGGTGAAG | | | ATGATGATGATGATGCTCGAGTCGACTTAGTCTCCCACCCTTTC |
| For expression analysis | | | | |
| OsLMP1-F/R | GCAGAAGGAAGCGAATGAAG | | | ATTTCTTGAACCGACCATGC |
| For CHIP-qPCR | | | | |
| P1 | GATGGTTGGTGGGTCTTCT | | | TTATTGTCTCGTTGGGTTTCA |
| P2 | ACAATAAAGAAGGGCGGC | | | ATAAGTCCCGTCAAACGC |
| P3 | TGTATTGCGCAGAACGAAAG | | | GTAGGTGATGGGCTGATGGT |
| P4 | ACCACCACGAAACCTCTACG | | | TTTAAATAGGAGGCGCAGGA |
| P5 | CATCCTGCGCCTCCTATTTA | | | CAGAGGGGAGATCGATGTGT |
| P6 | CTGGTGAGAGCTTGTGTGGA | | | CCCTGGAGCAGAGTGTTGAT |
| P7 | CTCCCGTCAGCCCAATAGTA | | | GGGGGTGGTTACAGGTTCTT |
| P8 | TCTGAACCACCACGTTTCAA | | | GGGGGTGGTTACAGGTTCTT |
| P9 | ATTGTTACTACATGTATGT | | | CTTTTCCGTC ACATCGTTC |
| P10 | GCTACTGCTTGCGACTCG | | | GGATTGCGTGCTGCTCTT |
| P11 | CGGCAGGAAGGTGAACGC | | | GGGTGAGGTGGTCGGTGT |
| P12 | GCAACTTCCAGGGCACCC | | | CTCGGAGCAGTAGGACGC |
| P13 | CGAGCCAAACAAGTCGTC | | | CGAATGTGGAGATAAACCCA |
| P14 | GGGAGATTCTGGTTGCCA | | | ACGCTCTGCGATTATTCAC |
| P15 | TGTCCCTCCTTCACTTTCC | | | CGAGTAATCTAACCAACCTGA |
| P16 | TAATGTAGTGATGTTCAAGTGC | | | TCTCTTCTCCCTCTGCCT |
| P17 | CCTTACCTACCTACACCCG | | | CACGAGGAGAAGAGAGGAT |
| P18 | ACGGCAGGAAGGTGGACG | | | TGTGGGTCAGGTGGTCGG |
| P19 | CCGTGTTTGCTATGGAGAC | | | GCCTAACTCTTCATCAGCC |
| P20 | ATAAGCAGGCAATACGGTG | | | AAATGCGACAAAGGTCCAT |
| P21 | CCAAGATGATACCAAAGGACG | | | GAGAGGAGAGGAGAGGAGAA |
| P22 | CCCTTCTCTTGTGGGATTAGA | | | TGTGGTCGGTCTGGTAGT |
| P23 | CAGGTCTCGCTTGTGCCT | | | TCAAACCACCAATCAGTTGC |
| P24 | TTGCCCTTGGTATGCTTAC | | | TTTCATCAATGCTTCTCTTAGC |
